# Major dietary patterns in relation to disease severity, symptoms, and inflammatory markers in patients recovered from COVID-19

**DOI:** 10.3389/fnut.2022.929384

**Published:** 2022-08-22

**Authors:** Armin Ebrahimzadeh, Mohsen Taghizadeh, Alireza Milajerdi

**Affiliations:** Research Center for Biochemistry and Nutrition in Metabolic Diseases, Institute for Basic Sciences, Kashan University of Medical Sciences, Kashan, Iran

**Keywords:** dietary patterns, COVID-19, inflammation, symptoms, disease severity

## Abstract

**Background:**

COVID-19 is a highly transmissible viral infection with high morbidity. Few studies have been done about dietary intakes in patients with COVID-19. This study aimed to evaluate the association between major dietary patterns before COVID-19 diagnosis in recovered patients and the risk of disease severity and symptoms after the disease begins.

**Methods:**

Overall, 250 recovered cases with both genders completed study questionnaires providing data on demographic characteristics, self-reported web-based 168-item food frequency questionnaire (FFQ), and COVID-19 outcomes in Shahid Beheshti Hospital, Kashan. PCR was used to determine a positive diagnosis of COVID-19. We used multivariable logistic regression models to assess the association between major dietary patterns and study outcomes. All statistical analyses were done by SPSS version 16.

**Results:**

We identified three major dietary patterns—unhealthy, traditional, and healthy dietary patterns. Serum levels of C-reactive protein (CRP) and erythrocyte sedimentation rate (ESR) were significantly higher in patients with unhealthy and traditional dietary patterns and lower in those with healthy dietary patterns. There was a significant direct relationship between unhealthy and traditional patterns with risk of severe COVID-19 and hospitalization duration and a significant direct association between an unhealthy pattern and the odds ratio (OR) of convalescence duration. A significant inverse relationship was found between healthy pattern and risk of severe COVID-19 and OR of convalescence duration. We found a significant direct association between unhealthy pattern and OR of cough, fever, chilling, weakness, myalgia, nausea and vomiting, and sore throat and between traditional pattern and OR of cough, fever, and chilling. In contrast, a significant inverse association was seen between healthy pattern and OR of dyspnea, weakness, and sore throat.

**Conclusion:**

This study showed that high adherence to an healthy pattern was associated with lower CRP and ESR levels and lower risk of severe COVID-19, hospitalization, and convalescence duration in patients who recovered from COVID-19. More adherence to unhealthy or traditional dietary patterns was associated with higher CRP and ESR levels and a higher risk of severe COVID-19 and hospitalization duration. A direct association was found between unhealthy and traditional patterns and the risk of some COVID-19 symptoms, while an inverse association was found for a healthy dietary pattern.

## Introduction

Coronaviruses are a large family of viruses that can cause respiratory infections in animals and humans (1). COVID-19 is an infectious disease caused by a new coronavirus and was first observed in Wuhan, China (2). The disease was unknown before it began to spread in Wuhan in December 2019 (2). So far, it has affected more than 190 countries around the world (3, 4). The clinical and laboratory features of COVID-19 are similar to the severe acute respiratory syndrome (SARS), which was first observed in China, and Middle East respiratory syndrome (MERS), which was first observed in Saudi Arabia (5).

Acute respiratory syndrome in COVID-19 is the main cause of hospitalization in the intensive care unit (ICU) and death (6). Cytokine storm is the main cause of organ dysfunction among these patients (7). Among environmental factors, dietary intake is an important factor affecting inflammation in the body (8–10). Therefore, it seems that the dietary intake of patients with COVID-19 before the beginning of the disease might influence the disease outcomes (11). So far, numerous studies have indicated that deficiency in vitamins and minerals might influence susceptibility to infectious diseases (12). In addition, some studies have shown the special role of some vitamins such as vitamin D in the immune function through infectious diseases (13, 14). However, it must be kept in mind that interactions between nutrients might confound the association of a specific nutrient with COVID-19. Therefore, the dietary pattern can be used as a new direction in nutritional epidemiology to find diet–disease relationships.

A recent population-based case–control study among six countries indicated that consumption of a plant-based diet was associated with a lower odds ratio (OR) of moderate to severe COVID-19 (15). Another study about COVID-19 symptoms and habitual food intake in adult outpatients indicated that an increase in habitual intake of legumes and grains, bread, and cereals was associated with reduced overall symptom severity in patients with COVID-19 (11). A recent study about diet and duration of recovery from COVID-19 showed that adherence to a healthy diet was associated with a shorter duration of recovery from COVID-19 (16).

Although the association of several nutrients with COVID-19 outcomes has received great attention, we are unaware of any study linking major dietary patterns to COVID-19 outcomes. Therefore, this study is conducted to determine the relationship between major dietary patterns and COVID-19 outcomes in Kashan, Iran.

## Methods

This retro-prospective study was performed among 250 recovered cases of COVID-19 aged 18–65 years of both genders, who were selected using a simple random sampling method from Shahid Beheshti Hospital, Kashan, Iran. This study was performed from June to September 2021. The study protocol was approved by the Ethics Committee of Kashan University of Medical Sciences (Registration No. IR.KAUMS.MEDNT.REC.1400.048). All participants were requested to complete informed consent.

**Figure 1:**
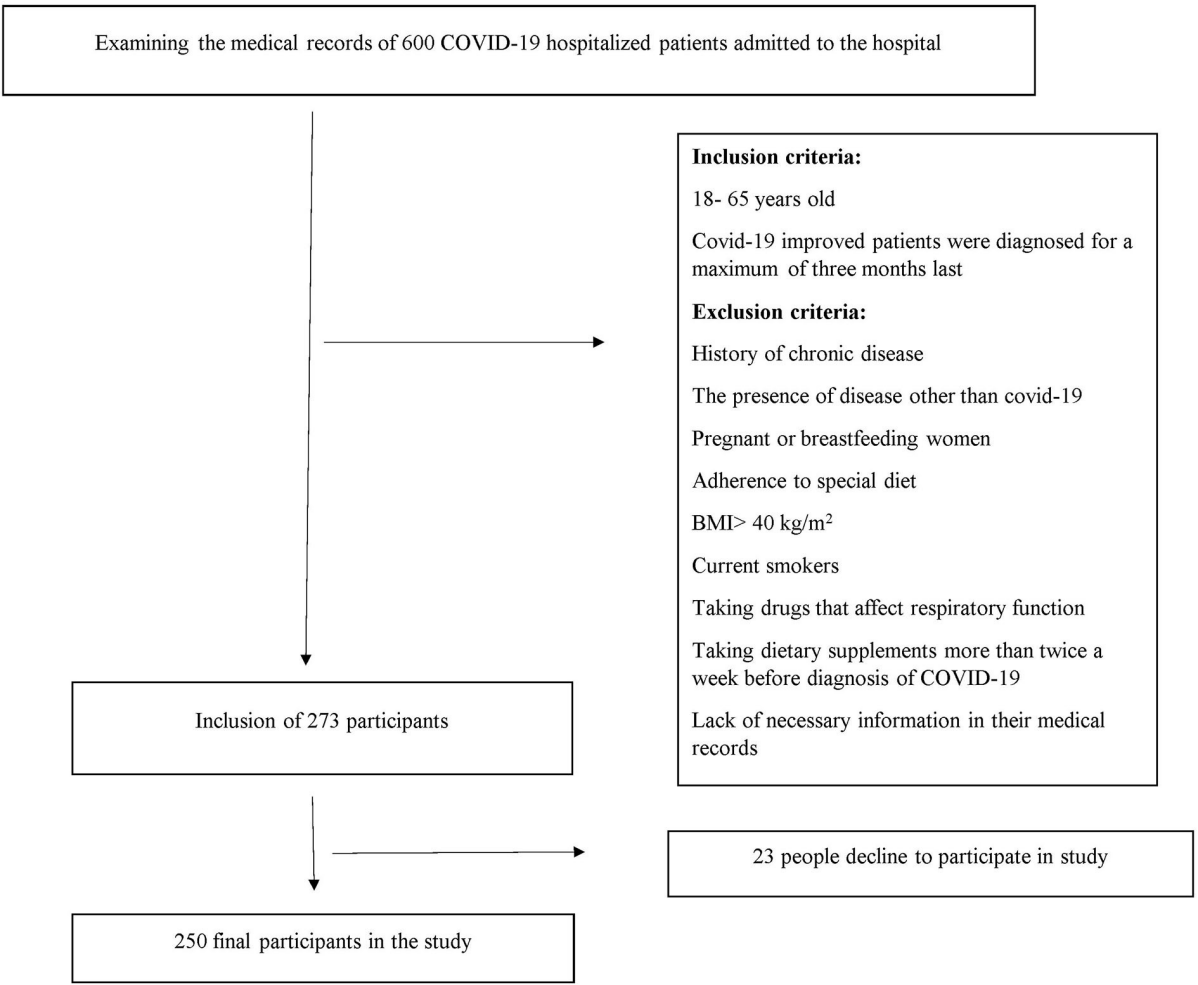
Flowchart of the study's participants.

All patients with COVID-19 who had medical records in the Shahid Beheshti Hospital with a maximum of 3 months from the beginning of their COVID-19 diagnosis were included. Patients were excluded if any of the following conditions existed: 1- Patients with other diseases than COVID-19. 2- those who had a history of chronic diseases such as diabetes and heart disease as well as diseases that affect the severity of COVID-19. 3- patients with a body mass index of more than 40. 4- pregnant or breastfeeding women; 5- current smokers. 6- patients who were consuming dietary supplements more than two times in a week before the first diagnosis of COVID-19. 7- patients who were on specific diets. 8- patients who were consuming medicines that influence respiratory function including fluticasone and flunisolide; and 9- subjects with insufficient data in their medical records (Figure 1).

### Assessment of dietary intake

A 168-item food frequency questionnaire (FFQ) was obtained from patients through a web-based online questionnaire to collect information on their dietary intakes during the past year before the diagnosis of COVID-19. Participants were asked to report their dietary intakes as daily, monthly, or annually. Finally, we converted their intakes of food items into grams per day using “household measures.” Dietary intakes of micro- and macro-nutrients were calculated by the use of the Nutritionist 4 (N4) software.

### Measurement of COVID-19 severity

COVID-19 severity was assessed by the COVID-19 Treatment Guidelines (CTG) (17), updated on 19 October 2021. According to the CTG, the severity of COVID-19 was categorized into five levels. Asymptomatic or presymptomatic infection: individuals with a positive test for SARS-CoV-2 using a virologic test (i.e., a nucleic acid amplification test [NAAT] or an antigen test) but without the symptoms of COVID-19. Mild illness: individuals with any of the various signs and symptoms of COVID-19 (e.g., fever, cough, sore throat, malaise, headache, muscle pain, nausea, vomiting, diarrhea, and loss of taste and smell) but without breath shortness, dyspnea, or abnormal chest imaging. Moderate illness: individuals with evidence of lower respiratory disease during clinical assessment or imaging and who have an oxygen saturation (SpO2) ≥94% on room air at sea level. Severe illness: individuals with SpO2 <94% on room air at sea level, a ratio of arterial partial oxygen pressure to the fraction of inspired oxygen (PaO2/FiO2) <300 mm Hg, and a respiratory rate >30 breaths/min or lung infiltrates >50%. Critical illness: individuals who had respiratory failure, septic shock, and/or multiple organ dysfunctions. We considered mild and moderate illnesses as a non-severe illness.

### Measurement of COVID-19 symptoms

We asked patients to fulfill a general questionnaire including a question about the presence of each common symptom of COVID-19. These symptoms were dyspnea, cough, fever, chilling, weakness, myalgia, sore throat, nausea, and vomiting.

### Assessment of inflammatory markers

Data on erythrocyte sedimentation rate (ESR) and C-reactive protein (CRP) were obtained from medical records. First measurements of CRP and ESR at the beginning of the disease were obtained.

### Assessment of other variables

Required information on demographic characteristics, physical activity, convalescence duration, supplements intake, corticosteroids use, antiviral drug use, and participants' height and weight were obtained for each subject by a general questionnaire.

### Statistical analysis

Normal distribution of data was explored by the Kolmogorov-Smirnov test. We classified 168 food items in FFQ into 21 predefined food groups to identify dietary patterns. The similarity of nutrients in food items was the basis for this classification: eggs, processed meat, sweets and desserts, sweetened drinks, meats, solid oils, junk foods, liquid oils, salt, refined grains, whole grains, flavors and pickles, chicken and fish, caffeine-containing drinks, red meat, vegetables, fruits and juices, low-fat dairy products, nuts, high-fat dairy products, and legumes (18–20). We conducted varimax rotation to generate a simple and differential varimax. The scree plot test and eigenvalues>1 were used to determine major dietary patterns. Adherence score for each dietary pattern was obtained and participants were categorized as tertiles based on these scores. We used an independent *t*-test to compare quantitative variables between categories of dietary patterns. A chi-square test was used to compare qualitative variables between categories. The correlation between adherence to each dietary pattern with outcomes of interest by considering confounding variables was assessed by the multivariable regression test. All statistical analyses were performed by the Statistical Package for Social Sciences software (SPSS Inc., version 16). A *p*-value of <0.05 was considered statistically significant.

## Results

### Major dietary patterns

We identified three major dietary patterns – unhealthy, traditional, and healthy dietary patterns (21). The unhealthy pattern was mainly characterized by a high intake of processed meats, sweets and desserts, energy drinks, red meats, solid oils, and junk foods. Participants in the traditional pattern had a high load of sweets and desserts, solid oils, salt, refined cereals, caffeine, and high-fat dairy product consumption. The healthy pattern was highly characterized by the intake of eggs, liquid oils, flavors and pickles, chicken and fish, vegetables, fruits and fruit juices, and nuts and legumes. Factor loadings of food groups in these major patterns are shown in Table 1.

**Table 1:** Factor loadings of food groups in major dietary patterns.

**Food group**	**Major dietary pattern**		
	**Unhealthy**	**Traditional**	**Healthy**
Egg			0.427
processed meat	0.726		
Sweets and desserts	0.543	0.466	
Energy drinks	0.833		
Visceral meats			
Solid oils	0.702	0.423	
Junk foods	0.679		
liquid oils			0.550
Salt	0.459	0.526	
Refined cereal		0.657	
Whole grain		−0.684	
Flavor and pickle	0.482		0.541
Chicken and fish	−0.606		0.555
Caffeine		0.579	
Red meats	0.432		
Vegetables	−0.675		0.461
Fruits and juice	−0.702		0.439
Low fat dairy products	−0.471	−0.445	
Nuts			0.765
High fat dairy products		0.521	
Legume	−0.561		0.421

### Characteristics of participants according to tertiles of major dietary patterns

The characteristics of participants according to tertiles of major dietary patterns are shown in Table 2. We found significant differences in age (43.80 ± 11.52 vs. 47.26 ± 11.33, *p* = <0.01), body mass index (BMI) (29.27 ± 3.56 vs. 26.20 ± 3.01, *p* = <0.001), likelihood of overweight or obesity (75 vs. 51, *p* = <0.001), supplements intake (83 vs. 79, *p* = <0.01), and corticosteroids use and antiviral drug use (83 vs. 77, *p* = <0.01) in the highest vs. lowest tertiles of unhealthy dietary pattern. However, there were no significant differences in physical activity level and gender between tertiles of the unhealthy pattern. With regard to traditional pattern, significant differences were observed in age (43.04 ± 12.41 vs. 42.21 ± 12.28, *p* = 0.02), BMI (27.96 ± 3.48 vs. 25.16 ± 2.77, *p* = <0.001), likelihood of overweight or obesity (64 vs. 46, *p* = 0.01), and corticosteroids use and antiviral drug use (81 vs. 70, *p* = <0.001) between the highest and lowest tertiles of the traditional pattern. There were no significant differences in physical activity level, gender, and percentage of participants with supplement use across tertiles of the dietary pattern. Finally, significant differences were observed in age (41.62 ± 12.46 vs. 46.49 ± 11.66, *p* = 0.03), BMI (26.24 ± 3.62 vs. 27.95 ± 3.4, *p* = <0.01), and the likelihood of overweight or obesity (47 vs. 65, *p* = 0.01) between the highest and lowest tertiles of healthy pattern, while no significant differences were found in physical activity level, gender, supplements intake, and corticosteroids or antiviral drug use.

**Table 2:** General characteristics of participants across tertiles of dietary patterns.

**Variables**		**Dietary patterns**								
		**Unhealthy**			**Traditional**			**Healthy**		
		**T1**	**T3**	***p*–value**	**T1**	**T3**	***p*–value**	**T1**	**T3**	***p*–value**
Age (year)		47.26 ± 11.33	43.80 ± 11.52	<0.01	42.21 ± 12.28	43.04 ± 12.41	0.02	46.49 ± 11.66	41.62 ± 12.46	0.036
BMI (kg/m^2^)		26.20 ± 3.01	29.27 ± 3.56	<0.001	25.16 ± 2.77	27.96 ± 3.48	<0.001	27.95 ± 3.4	26.24 ± 3.62	<0.01
Physically active (%)	sedentary	11(13.3)	11(13.3)	0.932	6(7.2)	9(10.8)	0.171	12(14.5)	6(7.2)	0.157
	moderate	67(80.7)	65(78.3)		71(85.5)	67(80.7)		64(77.1)	69(83.1)	
	intense	5(6)	7(8.4)		6(7.2)	7(8.4)		7(8.4)	8(9.6)	
Gender (female) (%)		42(50.6)	44 (53.0)	0.93	45(54.2)	44(53.0)	0.81	48(57.8)	37(44.6)	0.21
Overweight or obese (%)		51 (61.4)	75 (90.4)	<0.001	46 (55.4)	64 (77.1)	0.01	65 (78.3)	47 (56.6)	0.012
Supplements intake (%)		79 (95.2)	83 (100)	<0.01	75 (90.4)	81 (97.6)	0.08	78 (94)	77 (92.8)	0.34
Corticosteroids use (%)		77 (92.8)	83 (100)	<0.01	70 (84.3)	81 (97.6)	<0.001	77 (92.8)	74 (89.2)	0.49
Antiviral drugs use (%)		77 (92.8)	83 (100)	<0.01	70 (84.3)	81 (97.6)	<0.001	77 (92.8)	74 (89.2)	0.49

### Dietary intake of nutrients across tertiles of major dietary patterns

Dietary intake of nutrients across tertiles of major dietary patterns is shown in Table 3. Comparing the highest to the lowest tertile of unhealthy pattern, significant differences were observed in total energy (2926.94 ± 447.75 vs. 2653.89 ± 429.51, *p* = <0.001), carbohydrate (431.69 ± 43.66 vs. 408.33 ± 54.36, *p* = <0.001), fat (120.83 ± 21.49 vs. 87.65 ± 20.69, *p* = <0.001), dietary fiber (19.44 ± 1.8 vs. 26.57 ± 3.9, *p* = <0.001), vitamins B_1_ (2.5 ± 0.3 vs. 2.56 ± 0.37, *p* = <0.01), B_6_ (1.59 ± 0.27 vs. 1.78 ± 0.3, *p* = <0.001), B_9_ (357.78 ± 40.25 vs. 466.44 ± 85.29, *p* = <0.001), B_12_ (3.72 ± 0.62 vs. 4.74 ± 1.24, *p* = <0.001), C (111.5 ± 13.5 vs. 165.5 ± 31.64, *p* = <0.001), E (8.5 ± 2.24 vs. 6.8 ± 1.6, *p* = <0.001), D (2.59 ± 0.72 vs. 2.01 ± 0.58, *p* = <0.001), and A (1,175 ± 166 vs. 1,438 ± 322, *p* = <0.001), and calcium (867.2 ± 76.08 vs. 951.11 ± 145.57, *p* = <0.001) and magnesium (306.79 ± 25.68 vs. 354.68 ± 55.12, *p* = <0.001) intakes. In addition, dietary intakes of energy (2,894.68 ± 416.27 vs. 2,586.39 ± 503.03, *p* = <0.001), carbohydrate (437.58 ± 45.31 vs. 385.43 ± 59.31, *p* = <0.001), fat (106.80 ± 23.60 vs. 90.6 ± 28.53, *p* = <0.001), vitamins D (2.45 ± 0.64 vs. 2.14 ± 0.79, *p* = 0.019), E (8.1 ± 1.99 vs. 5.8 ± 1.46, *p* = <0.001), and B_1_ (2.65 ± 0.3 vs. 2.3 ± 0.37, *p* = <0.001), and calcium (945.5 ± 134.4 vs. 906.7 ± 164.05, *p* = <0.01) were significantly different between the highest in contrast to the lowest tertile of traditional pattern. Comparing the highest to the lowest tertiles of healthy pattern, we found significant differences in total energy (2,892.26 ± 414.30 vs. 2,551.5 ± 529.97, *p* = <0.001), protein (121.23 ± 11.15 vs. 92.84 ± 15.86, *p* = <0.001), carbohydrate (422.07 ± 49.72 vs. 391.52 ± 62.2, *p* = <0.001), fat (108.41 ± 16.91 vs. 84.73 ± 30.8, *p* = <0.001), dietary fiber (26.38 ± 4.7 vs. 20.05 ± 4.4, *p* = <0.001), vitamins B_1_ (2.6 ± 0.32 vs. 2.35 ± 0.37, *p* = <0.001), B_6_ (1.9 ± 0.15 vs. 1.39 ± 0.25, *p* = <0.001), B_9_ (484.76 vs. 347.86 ± 85.53, *p* = <0.001), B_12_ (4.8 ± 0.91 vs. 3.39 ± 1.14, *p* = <0.001), C (162.04 ± 29.34 vs. 113.55 ± 31.36, *p* = <0.001), E (7.06 ± 1.4 vs. 6.64 ± 2.33, *p* = <0.01), D (2.32 ± 0.57 vs. 2.1 ± 0.97, *p* = 0.032), and A (1,461 ± 271 vs. 1,139 ± 281, *p* = <0.001), and zinc (11.6 ± 1.1 vs. 8.6 ± 1.6, *p* = <0.001), calcium (998.9 ± 111.8 vs. 823.93 ± 136.49, *p* = <0.001), and magnesium (370.16 ± 37 vs. 282.4 ± 49.23, *p* = <0.001) intakes.

**Table 3:** Daily nutrient intake of all subjects across tertiles of dietary patterns.

**Nutrients**	**Major dietary pattern**								
	**Unhealthy**			**Traditional**			**Healthy**		
	**T1**	**T3**	***p*–value**	**T1**	**T3**	***p*–value**	**T1**	**T3**	***p*–value**
Energy (Kcal/day)	2653.89 ± 429.51	2926.94 ± 447.75	<0.001	2586.39 ± 503.03	2894.68 ± 416.27	<0.001	2551.5 ± 529.97	2892.26 ± 414.30	<0.001
Protein (g/day)	111.59 ± 18.54	105.20 ± 11.12	0.052	106 ± 21.85	111.21 ± 15.36	0.122	92.84 ± 15.86	121.23 ± 11.15	<0.001
Carbohydrate (g/day)	408.33 ± 54.36	431.69 ± 43.66	<0.001	385.43 ± 59.31	437.58 ± 45.31	<0.001	391.52 ± 62.2	422.07 ± 49.72	<0.001
Fat (g/day)	87.65 ± 20.69	120.83 ± 21.49	<0.001	90.6 ± 28.53	106.80 ± 23.60	<0.001	84.73 ± 30.8	108.41 ± 16.91	<0.001
Dietary fiber (g/day)	26.57 ± 3.9	19.44 ± 1.8	<0.001	23.46 ± 4.44	23.33 ± 5.33	0.58	20.05 ± 4.4	26.38 ± 4.7	<0.001
Vitamin D	2.01 ± 0.58	2.59 ± 0.72	<0.001	2.14 ± 0.79	2.45 ± 0.64	0.019	2.1 ± 0.97	2.32 ± 0.57	0.032
Vitamin A	1438 ± 322	1175 ± 166	<0.001	1346 ± 424	1341 ± 274	0.299	1139 ± 281	1461 ± 271	<0.001
Vitamin E	6.8 ± 1.6	8.5 ± 2.24	<0.001	5.8 ± 1.46	8.1 ± 1.99	<0.001	6.64 ± 2.33	7.06 ± 1.4	<0.01
Vitamin C	165.5 ± 31.64	111.5 ± 13.5	<0.001	144.32 ± 30.94	138.14 ± 42.86	0.282	113.55 ± 31.36	162.04 ± 29.34	<0.001
Vitamin B_1_	2.56 ± 0.37	2.5 ± 0.3	<0.01	2.3 ± 0.37	2.65 ± 0.3	<0.001	2.35 ± 0.37	2.6 ± 0.32	<0.001
Vitamin B_6_	1.78 ± 0.3	1.59 ± 0.27	<0.001	1.69 ± 0.33	1.66 ± 0.32	0.222	1.39 ± 0.25	1.9 ± 0.15	<0.001
Vitamin B_9_	466.44 ± 85.29	357.78 ± 40.25	<0.001	426.53 ± 95.3	411.69 ± 96.76	0.47	347.86 ± 85.53	484.76	<0.001
Vitamin B_12_	4.74 ± 1.24	3.72 ± 0.62	<0.001	3.99 ± 1.5	4.28 ± 1.02	0.273	3.39 ± 1.14	4.8 ± 0.91	<0.001
Zinc	10.45 ± 1.74	10.01 ± 1.16	0.24	10.35 ± 2.37	10.28 ± 1.53	0.923	8.6 ± 1.6	11.6 ± 1.1	<0.001
Calcium	951.11 ± 145.57	867.2 ± 76.08	<0.001	906.7 ± 164.05	945.5 ± 134.4	<0.01	823.93 ± 136.49	998.9 ± 111.8	<0.001
Magnesium	354.68 ± 55.12	306.79 ± 25.68	<0.001	321.76 ± 60.5	336.83 ± 54.62	0.187	282.4 ± 49.23	370.16 ± 37	<0.001

### The association between major dietary patterns, CRP, and ESR

The association between major dietary patterns and inflammatory markers after adjustment for sex, age, BMI, physical activity, and energy intake in patients with COVID-19 is shown in Table 4. Serum levels of CRP and ESR were significantly higher in patients at top tertiles of unhealthy (35.62 ± 24.29 vs. 10.61 ± 14.85, *p* = <0.001 and 41.65 ± 29.83 vs. 15 ± 16, *p* = <0.001, respectively) and traditional (25.3 ± 24.44 vs. 12.63 ± 18.43, *p* = <0.001 and 31.33 ± 30.66 vs. 16.96 ± 16.57, *p* = <0.001, respectively) dietary patterns than those at the bottom. In contrast, our analysis indicated lower levels of CRP and ESR in those at the third tertile vs. those at the first tertile of the healthy dietary pattern (11.89 ± 12.42 vs. 26.14 ± 25.5, *p* = <0.001 and 15.62 ± 11.69 vs. 33.9 ± 29.88, *p* = <0.001, respectively).

**Table 4:** Inflammatory biomarkers across tertiles of dietary patterns.

**Dietary patterns**												
	**Unhealthy**				**Traditional**				**Healthy**			
	**T1**	**T3**	***P*** **–value**	**Ad.P**	**T1**	**T3**	***p*** **–value**	**Ad.P**	**T1**	**T3**	***p*** **–value**	**Ad.P**
CRP (mg/dl)	10.61 ± 14.85	35.62 ± 24.29	<0.001	<0.001	12.63 ± 18.43	25.3 ± 24.44	<0.01	<0.001	26.14 ± 25.5	11.89 ± 12.42	<0.001	<0.001
ESR (mm/hr)	15 ± 16	41.65 ± 29.83	<0.001	<0.001	16.96 ± 16.57	31.33 ± 30.66	<0.01	<0.001	33.9 ± 29.88	15.62 ± 11.69	<0.001	<0.001

Adjusted for sex, age, BMI, physical activity and energy intake.

### The relationship between major dietary patterns and the risk of severe COVID-19

Multivariable binary logistic regression for the relationship between major dietary patterns and risk of severe COVID-19 is indicated in Table 5. In the crude model, we found a significant direct association between adherence to unhealthy and traditional dietary patterns and risk of severe COVID-19 (OR: 4.34; 95% confidence interval (CI): 2.26, 8.34, *p* = <0.001 and 3.37; 95% CI: 1.77, 6.42, *p* = <0.001, respectively). Such relationship was also observed after controlling for age, sex, and energy intake (OR: 5.06; 95% CI: 2.51, 10.21, *p* = <0.001 and 3.61; 95% CI: 1.81, 7.19, *p* = <0.001, respectively). Additional adjustments for other potential confounders including physical activity, supplement use, corticosteroids use, and antiviral drug use had no effect on the association (OR: 4.57; 95% CI: 2.34, 9.64, *p* = <0.01 and 3.13; 95% CI: 1.55, 6.3, *p* = <0.01, respectively). In the fully adjusted model, this association also remained significant (OR: 3.23; 95% CI: 1.53, 6.81, *p* = <0.01 and 2.17; 95% CI: 1.03, 4.54, *p* = 0.04, respectively). We found a significant inverse relationship between adherence to healthy pattern and risk of severe COVID-19 (OR: 0.25; 95% CI: 0.13, 0.49, *p* = <0.001). After adjustments for the potential confounders in three models, the association remained as statistically significant (model 1: 0.22, 95% CI: 0.1, 0.45, *p* = <0.001; model 2: 0.21, 95% CI: 0.1, 0.45, *p* = <0.01; model 3: 0.31, 95% CI: 0.14, 0.68, *p* = <0.01).

**Table 5:** Odds ratio (95% CI) of severe disease in relation to major dietary patterns.

	**Dietary patterns**								
	**Unhealthy**			**Traditional**			**Healthy**		
	**T1**	**T3**	***p*–value**	**T1**	**T3**	***p*–value**	**T1**	**T3**	***p*–value**
Crude	1	4.34 (2.26, 8.34)	<0.001	1	3.37 (1.77, 6.42)	<0.001	1	0.25 (0.13, 0.49)	<0.001
Model 1	1	5.06 (2.51, 10.21)	<0.001	1	3.61 (1.81, 7.19)	<0.001	1	0.22 (0.1, 0.45)	<0.001
Model 2	1	4.57 (2.34, 9.64)	<0.01	1	3.13 (1.55, 6.3)	<0.01	1	0.21 (0.1, 0.45)	<0.01
Model 3	1	3.23 (1.53, 6.81)	<0.01	1	2.17 (1.03, 4.54)	0.04	1	0.31 (0.14, 0.68)	<0.01

Model 1, Adjusted for sex, age and energy intake; Model 2, Further adjusted for physical activity, supplement use, corticosteroids use, and antiviral drugs use; Model 3, Further adjusted for BMI.

### The association between major dietary patterns and the risk of each COVID-19 symptom

Multivariable binary logistic regression for the association between major dietary patterns and the risk of each COVID-19 symptom is shown in Table 6. After controlling for potential confounders, we found a significant direct association between unhealthy pattern and OR of cough (6.21, 95% CI: 2.5, 15.45, *p* = <0.01), fever (9.07, 95% CI: 2.83, 28.98, *p* = <0.001), chilling (12.21, 95% CI: 3.34, 44.64, *p* = <0.001), weakness (2.25, 95% CI: 1.01, 5, *p* = 0.04), myalgia (2.91, 95% CI: 1.41, 6, *p* = <0.01), nausea and vomiting (5.71, 95% CI: 1.74, 18.71, *p* = <0.01), and sore throat (9.6, 95% CI: 3.88, 23.77, *p* = <0.001). A significant direct association was also found between traditional pattern and OR of cough (2.79, 95% CI: 1.32, 5.92, *p* = <0.01), fever (2.36, 95% CI: 1.03, 5.39, *p* = 0.03), and chilling (2.55, 95% CI: 1.1, 5.92, *p* = 0.02) at the fully adjusted model. In contrast, a significant inverse association was seen between healthy pattern and OR of dyspnea (0.27, 95% CI: 0.11, 0.63, *p* = <0.01), weakness (0.22, 95% CI: 0.1, 0.51, *p* = <0.001), and sore throat (0.36, 95% CI: 0.16, 0.8, *p* = 0.01) at the third model of adjustment.

**Table 6:** Odds ratio (95% CI) for symptoms of COVID−19 according to major dietary patterns.

	**Dietary patterns**								
	**Unhealthy**			**Traditional**			**Healthy**		
	**T1**	**T3**	***p*–value**	**T1**	**T3**	***p*–value**	**T1**	**T3**	***p*–value**
**Dyspnea**									
Crude	1	3.37 (1.83, 7.75)	<0.001	1	3.2 (1.69, 6.19)	<0.001	1	0.28 (0.14, 0.55)	<0.001
Model 1	1	3.8 (1.81, 8.2)	<0.01	1	3.25 (1.62, 6.52)	<0.01	1	0.21 (0.1, 0.46)	<0.001
Model 2	1	3.46 (1.61, 7.4)	<0.01	1	2.79 (1.36, 5.7)	<0.01	1	0.2 (0.08, 0.45)	<0.001
Model 3	1	2.4 (1.08, 5.36)	0.53	1	2.07 (0.98, 4.37)	0.51	1	0.27 (0.11, 0.63)	<0.01
**Cough**									
Crude	1	8.31 (3.56, 19.38)	<0.001	1	4.04 (2.09, 7.82)	<0.001	1	0.44 (0.23, 0.83)	0.01
Model 1	1	8.74 (3.62, 21.08)	<0.001	1	4.05 (2.02, 8.1)	<0.001	1	0.35 (0.17, 0.7)	<0.01
Model 2	1	8.7 (3.61, 21.32)	<0.001	1	3.83 (1.89, 7.79)	<0.001	1	0.36 (0.17, 0.73)	<0.01
Model 3	1	6.21 (2.5, 15.45)	<0.01	1	2.79 (1.32, 5.92)	<0.01	1	0.55 (0.259, 1.19)	0.15
**Fever**									
Crude	1	10.6 (3.52, 31.9)	<0.001	1	3.21 (1.53, 6.73)	<0.01	1	1 (0.49, 2.36)	0.85
Model 1	1	11.44 (3.71, 35.29)	<0.001	1	3.24 (1.49, 7.08)	<0.01	1	1.1 (0.47, 2.57)	0.79
Model 2	1	10.92 (3.52, 33.8)	<0.001	1	2.9 (1.3, 6.47)	<0.01	1	0.65 (1.22, 0.51	0.65
Model 3	1	9.07 (2.83, 28.98)	<0.001	1	2.36 (1.03, 5.39)	0.03	1	1.71 (0.69, 4.27)	0.17
**Chilling**									
Crude	1	14.32 (4.15, 49.37)	<0.001	1	3.52 (1.65, 7.5)	<0.01	1	1 (0.45, 2.2)	1
Model 1	1	16.43 (4.63, 58.21)	<0.001	1	3.65 (1.65, 8.07)	<0.01	1	1.06 (0.45, 2.47)	0.85
Model 2	1	15.68 (4.41, 55.73)	<0.001	1	3.26 (1.44, 7.36)	<0.01	1	1.16 (0.48, 2.78)	0.71
Model 3	1	12.21 (3.34, 44.64)	<0.001	1	2.55 (1.1, 5.92)	0.02	1	1.75 (0.69, 4.43)	0.15
**Weakness**									
Crude	1	2.69 (1.35, 5.36)	<0.01	1	0.88 (0.44, 1.75)	0.73	1	0.21 (0.1, 0.44)	<0.001
Model 1	1	2.96 (1.42, 6.19)	<0.01	1	0.75 (0.36, 1.57)	0.45	1	0.19 (0.08, 0.41)	<0.001
Model 2	1	3.08 (1.44, 6.56)	<0.01	1	0.73 (0.34, 1.56)	0.41	1	0.18 (0.08, 0,41)	<0.001
Model 3	1	2.25 (1.01, 5)	0.04	1	0.5(0.22, 1.12)	0.087	1	0.22 (0.1, 0.51)	<0.001
**Myalgia**									
Crude	1	0.25 (0.13, 0.48)	<0.001	1	1.05 (0.56, 1.96)	0.87	1	0.47 (0.25, 0.88)	0.019
Model 1	1	3.63 (1.83, 7.2)	<0.001	1	0.95 (0.48, 1.85)	0.87	1	0.47 (0.24, 0.95)	0.03
Model 2	1	3.5 (1.76, 6.99)	<0.001	1	0.85 (0.43, 1.68)	0.632	1	0.49 (0.24, 0.97)	0.04
Model 3	1	2.91 (1.41, 6)	<0.01	1	0.61 (0.29, 1.26)	0.182	1	0.64 (0.31, 1.3)	0.23
**Nausea and vomiting**									
Crude	1	0.15 (0.05, 0.43)	<0.001	1	2.6 (0.94, 7.1)	0.07	1	0.24 (0.08, 0.711)	<0.01
Model 1	1	7.25 (2.31, 22.69)	<0.001	1	1.06 (0.458, 2.49)	0.17	1	0.16 (0.05, 0.49)	<0.01
Model 2	1	6.84 (2.18, 21.47)	<0.001	1	1.88 (0.66, 5.37)	0.29	1	0.16 (0.05, 0.16)	<0.01
Model 3	1	5.71 (1.74, 18.71)	<0.01	1	1.29 (0.43, 3.89)	0.75	1	0.22 (0.07, 0.71)	0.48
**Sore throat**									
Crude	1	9.2 (4.1, 20.96)	<0.001	1	1 (0.5, 1.99)	1	1	0.3 (0.14, 0.63)	<0.01
Model 1	1	11.08 (4.71, 26)	<0.001	1	0.9 (0.44, 1.86)	0.76	1	0.27 (0.12, 0.58)	<0.01
Model 2	1	12.3 (5.1, 29.8)	<0.001	1	0.92 (0.44, 1.9)	0.78	1	0.26 (0.12, 0.58)	<0.01
Model 3	1	9.6 (3.88, 23.77)	<0.001	1	0.62 (0.28, 1.37)	0.2	1	0.36 (0.16, 0.8)	0.01

Model 1, Adjusted for sex, age and energy intake; Model 2, Further adjusted for physical activity, supplement use, corticosteroids use, and antiviral drugs use; Model 3, Further adjusted for BMI.

### The association of dietary patterns and OR of lasted hospitalization and convalescence duration

Multivariable binary logistic regression for the association of each dietary pattern and OR of lasted hospitalization and convalescence duration is presented in Table 7. There were significant associations between unhealthy and traditional patterns with OR of long-term hospitalization after adjusting for confounder variables (2.87, 95% CI: 1.28, 6.45, *p* = 0.01 and 2.97, 95% CI: 1.23, 7.15, *p* = 0.01, respectively). After controlling for the potential confounders, there was no significant association between healthy pattern and OR of lasted hospitalization (0.44, 95% CI: 0.18, 1.06, *p* = 0.07). Furthermore, we did not find a significant association between the traditional pattern and OR of convalescence duration after adjustment for the potential confounders (1.04, 95% CI: 0.51, 2.11, *p* = 0.9). However, a significant direct association was found between the unhealthy pattern and OR of convalescence duration in the final model (1.06, 95% CI: 0.97, 1.16, *p* = <0.01). Moreover, a significant inverse association was seen between healthy pattern and OR of convalescence duration (0.32, 95% CI: 0.15, 0.68, *p* = <0.01).

**Table 7:** Odds ratio (95% CI) for hospital stay and convalescence duration according to major dietary patterns.

	**Dietary patterns**								
	**Unhealthy**			**Traditional**			**Healthy**		
	**T1**	**T3**	***p*–value**	**T1**	**T3**	***p*–value**	**T1**	**T3**	***p*–value**
**Hospital duration (days)**									
Crude	1	2.74 (1.42, 5,29)	<0.01	1	3.59 (1.74, 7.39)	<0.01	1	0.39 (0.19, 0.77)	<0.01
Model 1	1	3.65 (1.69, 7.92)	<0.01	1	4.04 (1.75, 9.34)	<0.01	1	0.37 (0.16, 0.84)	0.017
Model 2	1	3.23 (1.49, 7)	<0.01	1	3.64 (1.55, 8.57)	<0.01	1	0.36 (0.15, 0.83)	0.016
Model 3	1	2.87 (1.28, 6.45)	0.015	1	2.97 (1.23, 7.15)	0.011	1	0.44 (0.18, 1.06)	0.068
**Convalescence duration (days)**									
Crude	1	2.7 (1.38, 5.29)	<0.01	1	1.22 (0.65, 2.26)	0.52	1	0.24 (0.12, 0.47)	<0.01
Model 1	1	3.48 (1.7, 7.1)	<0.01	1	1.3 (0.67, 2.5)	0.44	1	0.26 (0.13, 0.53)	<0.01
Model 2	1	3.64 (1.77, 7.48)	<0.01	1	1.35 (0.69, 2.65)	0.39	1	0.25 (0.12, 0.52)	<0.01
Model 3	1	1.06 (0.97, 1.16)	<0.01	1	1.04 (0.51, 2.11)	0.91	1	0.32 (0.15, 0.68)	<0.01

Model 1, Adjusted for sex, age and energy intake; Model 2, Further adjusted for physical activity, supplement use, corticosteroids use, and antiviral drugs use. Model 3, Further adjusted for BMI.

## Discussion

This study aimed to evaluate the association between major dietary patterns before COVID-19 diagnosis in recovered patients and the risk of disease severity and symptoms after the disease begins.

More adherence to the healthy dietary pattern was associated with lower concentrations of CRP and ESR. In contrast, more adherence to unhealthy and traditional patterns was associated with higher concentrations of those pro-inflammatory markers. To the best of our knowledge, there is no study investigating the relationship between dietary patterns and inflammatory markers in patients with COVID-19. Findings from a case–control study showed a positive association between the dietary inflammatory index and serum levels of CRP and ESR in patients with COVID-19 (22). Other studies evaluated the relationship between an individual nutrient and the levels of CRP and ESR. For example, a meta-analysis suggested that consumption of healthy foods rich in antioxidant vitamins and phytochemicals was associated with lower CRP levels in men (23).

Our study indicated a positive relationship between unhealthy and traditional dietary patterns and the risk of severe COVID-19, while an inverse association was found for the healthy dietary pattern. A cross-sectional study on 236 patients with COVID-19 showed that an increment in habitual intake of legumes, grains, and bread and cereals decreased overall symptom severity in patients with COVID-19 (11). Moreover, a population-based case–control study in six countries indicated that individuals who consumed plant-based diets with a higher intake of vegetables, legumes, and nuts, and a lower intake of poultry and red or processed meats had lower OR of severe COVID-19-like illness (15). Furthermore, the results of a prospective cohort study supported the finding of our study, in which adherence to plant-based foods was associated with lower risk and severity of COVID-19 (24). Due to common food items in such dietary patterns, it seems that more detailed studies are needed to illustrate which food items are more important and which are the main anti-COVID-19 micronutrients or bioactive components in those foods.

With regard to the symptoms of COVID-19, we found a significant increment in the risk of dyspnea, cough, fever, chilling, weakness, myalgia, nausea and vomiting, and sore throat with more adherence to the unhealthy pattern. Adjustment for BMI disappeared the association for dyspnea. It seems that BMI is an important confounding factor in the relationship between dietary intake and the risk of COVID-19 symptoms. A recent systematic review and meta-analysis including 46 studies involving 625,153 patients indicated a greater risk of infection, hospitalization, clinically severe disease, mechanical ventilation, ICU admission, and mortality due to COVID-19 in patients with obesity (25). A significant positive relation was found between the traditional dietary pattern and dyspnea, cough, fever, and chilling. The association for dyspnea disappeared after adjustment for BMI. A lack of significant association for some symptoms might be due to the effect of other confounding factors that need to be taken into account in future investigations. For instance, patients' physical activity before COVID-19 diagnosis, their medical history, and family history of different diseases are among the most important confounders that should be attended to with more detail. In contrast to the two aforementioned dietary patterns, higher adherence to the healthy dietary pattern was associated with a lowered risk of dyspnea, weakness, and sore throat. To the best of our knowledge, this study is the first investigation into the association between dietary patterns and symptoms of COVID-19. A recent case–control study indicated that more intake of legumes, grains, and bread and cereals was associated with a reduction in overall symptom severity in patients with COVID-19 (11).

More adherence to unhealthy or traditional patterns was associated with increased duration of hospitalization in patients with COVID-19. Although an inverse association was seen between adherence to the healthy dietary pattern and hospitalization time in our study, the association was removed after additional adjustment for BMI in the third model. Similar to what we said for COVID-19 symptoms, it can be suggested that BMI is an important confounder in the relationship between dietary intake and hospital duration. Findings from a retrospective cohort study indicated that subjects with obesity who were affected by COVID-19 required longer hospitalization and more intensive and longer oxygen treatments (26).

Finally, we found a direct association between more adherence to the unhealthy dietary pattern and convalescence duration, while an inverse association was found for more adherence to the healthy dietary pattern. However, no significant association was found between the traditional dietary pattern and convalescence duration in patients with COVID-19. In line with our findings, a cross-sectional study on COVID-19 survivors in Saudi Arabia indicated that more adherence to a healthy diet was associated with a shorter duration of recovery from COVID-19 (16). Further studies about different common known dietary patterns are needed to expand the current finding.

The exact mechanisms through which dietary patterns might affect COVID-19 severity and symptoms are unknown. It is suggested that micro-nutrients in a diet might affect COVID-19 prognosis (27). Vitamin A has various functions in the body's immune system (28). Growth, development, and function of neutrophils, monocytes and macrophages, apoptosis, and gene expression of B and T lymphocytes are examples of vitamin A functions in the immune system (29). Vitamin B6 is another important factor in a diet that strengthens the immune system and increases the production of white blood cells including IL-2 and T cells (30). In addition, numerous studies have indicated the effective roles of vitamin C in the prevention of infections, such as SARS coronavirus (31, 32). A recent meta-analysis indicated that low serum vitamin D concentration was associated with more risk of in-hospital mortality among patients with COVID-19 (13). Roles of vitamin D in immune responses and protecting the body against various viruses have been reported previously (33).

For example, a recent meta-analysis indicated that vitamin D supplementation was associated with a reduction in the ICU admission rate, a reduction in the need for mechanical ventilation, and a reduction in mortality from COVID-19 (34).

Furthermore, vitamin E deficiency has been associated with lipid peroxidation (35), and omega-3 fatty acid has protective roles against infectious diseases by removing body inflammation (36). Cytokine storm in response to viral infections can lead to multi-organ failure in patients with COVID-19 (37). Furthermore, fibers are fermented by the gut flora to produce short-chain fatty acids, which have anti-inflammatory functions (38).

This study is the first investigation into the association of major dietary patterns with the risk of COVID-19 symptoms and severity. However, some limitations should also be taken into account when interpreting the findings of this study. This is a single-center study. Although the study population included adults, it would be prudent to consider their sample size and the fact that they were all drawn from the same center when determining their generalizability to the general population. We did not examine the socioeconomic status of participants, which may influence their dietary intake. Our study had a limited sample size, which highlights the need for larger studies. In addition, differences in virus variants can affect the severity and symptoms of COVID-19 (39, 40). Insufficient information in the medical records of some patients was another limitation of our study. Furthermore, we excluded patients with acute and very high severe diseases from our study. This was because of a lack of information about their dietary intake before the disease diagnosis and also due to their inability to fill out the questionnaires. We assessed the dietary intakes of participants with a self-reported web-based 168-FFQ. Therefore, recall bias and misclassification of participants by the dietary intakes should not be neglected. Finally, due to the cross-sectional design of this study, it is impossible to confer causality.

In conclusion, this study showed that high adherence to a healthy pattern was associated with less CRP and ESR and lower risk of severe COVID-19, and hospitalization and convalescence durations in patients who recovered from COVID-19. However, more adherence to unhealthy or traditional dietary patterns was associated with higher CRP and ESR, risk of severe COVID-19, and hospitalization duration. A direct association was found between adherence to the unhealthy pattern and risk of cough, fever, chilling, weakness, myalgia, nausea and vomiting, and sore throat, and between the traditional pattern with risk of dyspnea, cough, fever, and chilling. A healthy dietary pattern was inversely associated with the risk of dyspnea, cough, weakness, myalgia, nausea and vomiting, and sore throat.

## Data availability statement

The raw data supporting the conclusions of this article will be made available by the authors, without undue reservation.

## Ethics statement

The studies involving human participants were reviewed and approved by Ethics Committee of Kashan University of Medical Sciences. The patients/participants provided their written informed consent to participate in this study.

## Author contributions

AE: conceptualization, formal analysis, writing–original draft, writing–review and editing, and data collection. AM: supervision, conceptualization, methodology, investigation, funding acquisition, formal analysis, writing–original draft, and writing–review and editing. MT: supervision, conceptualization, formal analysis, writing–original draft, and writing–review and editing. All authors contributed to the article and approved the submitted version.

## Funding

This study was supported by the Kashan University of Medical Sciences in Kashan. This paper is extracted from the results of a research project (Registration No. IR.KAUMS.MEDNT.REC.1400.048), which was conducted at Shahid Beheshti Hospital, Kashan University of Medical Sciences.

## Conflict of interest

The authors declare that the research was conducted in the absence of any commercial or financial relationships that could be construed as a potential conflict of interest.

## Publisher's note

All claims expressed in this article are solely those of the authors and do not necessarily represent those of their affiliated organizations, or those of the publisher, the editors and the reviewers. Any product that may be evaluated in this article, or claim that may be made by its manufacturer, is not guaranteed or endorsed by the publisher.
