# Supplementary material for: Major dietary patterns in relation to disease severity, symptoms, and inflammatory markers in patients recovered from COVID-19
Source: Front Nutr. 2022 Aug 22;9:929384. doi: 10.3389/fnut.2022.929384 (PMC9446542; doi:10.3389/fnut.2022.929384)
Supplement: Supplementary file 1 [file Data_Sheet_1.PDF]

پرسشنامه بسامد خوراک

نام و نام خانوادگي:..

ردیف	مواد غذایی	مقدار	روز	هفته	ماه	سال	ملاحظات
۱	نان لواش	۱ کف دست					
۲	نان بربری	۱ کف دست					
۳	نان سنگک	۱ کف دست					
۴	نان تافتون	۱ کف دست					
۵	نان باگت	۱ عدد کوچک					
۶	نان تست(سبوس دار)	۱ کف دست					
۷	برنج پخته	۱ بشقاب غذاخوری					معمولی پر
۸	ماکارونی پخته	۱ کفگیر					سرصاف پر
۹	سیب زمینی	۱ عدد متوسط					
۱۰	سیب زمینی سرخ کرده	۱۰ خلال متوسط					
۱۱	ورمیشل پخته	۱ لیوان					
۱۲	رشته	۱ لیوان					
۱۳	آرد گندم	۱ استکان شستی					
۱۴	بیسکوئیت						نوع:....... اندازه:.......
۱۵	کراکر	۱ عدد					
۱۶	کیک یزدی	۱ عدد					
۱۷	کیک خانگی (تولد و..)	۱ برش متوسط					
۱۸	سایر کیک ها	۱ عدد					
۱۹	ذرت وبلال	۱ عدد متوسط					
۲۰	جو پخته	۱ ق.غ					
۲۱	بلغور پخته	۱ ق.غ					
۲۲	عدس	۱ ق.غ					
۲۳	لوبیا	۱ ق.غ					
۲۴	نخود	۱ ق.غ					
۲۵	باقلا پخته	۱ ق.غ					

۲۶	سویا	۱ ق.غ					
۲۷	ماش	۱ ق.غ					
۲۸	لپه	۱ ق.غ					
۲۹	گوشت گاو یا گوساله	۱ تکه خورشتی					
۳۰	گوشت گوسفند	۱ تکه خورشتی					
۳۱	گوشت چرخ کرده	۱ ق.غ					
۳۲	مرغ و جوجه با پوست	۱ قطعه متوسط					ران سینه بال
۳۳	مرغ و جوجه بدون پوست	۱ قطعه متوسط					ران سینه بال کباب
۳۴	ماهی (به استثنای کنسرو تن) با ذکر نوع						مقدار:.......<br>نوع:........
۳۵	تن ماهی (کنسرو)	۱/۲ قوطی					آیا روغن آن دور ریخته می شود؟ بله خیر
۳۶	همبرگر	۱ عدد					
۳۷	سوسیس	۱ عدد					کوکتل آلمانی
۳۸	کالباس	۱ برش					
۳۹	دل و جگر و قلوه	۱ تکه متوسط					
۴۰	تخم مرغ	۱ عدد					فقط سفیده فقط زرده
۴۱	سیرابی و شیردان	۱ قطعه					
۴۲	زبان	۱ عدد کامل					
۴۳	مغز	۱ عدد کامل					
۴۴	کله	۱ عدد کامل					
۴۵	پاچه	۱ عدد					
۴۶	پیتزا	۱ برش					
۴۷	شیر بی چرب	۱ لیوان					
۴۸	شیر کم چرب(۲%>)	۱ لیوان					
۴۹	شیرپر چرب(۲%<)	۱ لیوان					
۵۰	شیر کاکائو	۱ شیشه تجاری					
۵۱	شیر شکلاتی	۱ شیشه تجاری					
۵۲	ماست چکیده	۱ ق.غ					
۵۳	ماست معمولی	۱ کاسه ماست					

۵۴	ماست پر چرب	۱ کاسه ماست					
۵۵	ماست خامه ای	۱ ق.غ					
۵۶	پنیر (به استثنای خامه ای)	۱ قوطی کبریت					
۵۷	پنیر خامه ای	۱ قوطی کبریت					
۵۸	دوغ	۱ لیوان					
۵۹	خامه و سرشیر	۱ ق.غ					
۶۰	بستنی سنتی	نصف لیوان					
۶۱	بستنی غیرسنتی	نصف لیوان					
۶۲	کره (+ آنچه که به غذا افزوده می شود)	۱ قوطی کبریت					
۶۳	مارگارین (+ آنچه که به غذا افزوده می شود)	۱ قوطی کبریت					
۶۴	کشک	۱ ق.غ					
۶۵	کاهو خرد شده	نصف لیوان					خام پخته
۶۶	گوجه فرنگی	۱ عدد متوسط					
۶۷	خیار	۱ عدد متوسط					
۶۸	سبزی خوردن	۱ پیش دستی					
۶۹	سبزی خورشتی	نصف لیوان					
۷۰	کدو حلوایی	قطعه ۶×۶					
۷۱	کدو خورشتی	۱ عدد متوسط					
۷۲	بادمجان پخته	۱ عدد متوسط					سرخ شده دلمه ای
۷۳	کرفس پخته	نصف لیوان					
۷۴	نخود سبز پخته	نصف لیوان					
۷۵	لوبیا سبز پخته	۱ ق.غ					
۷۶	هویج خام	۱ عدد متوسط					
۷۷	هویج پخته	۱ عدد متوسط					
۷۸	سیر	۱ حبه					
۷۹	پیاز خام	۱ عدد متوسط					
۸۰	پیاز سرخ شده	۱ ق.غ					
۸۱	کلم (شامل کلم پیچ، کلم دگمه ای و گل کلم)	۱ کاسه ماست خوری					
۸۲	فلفل دلمه ای	۱ عدد متوسط					

۸۳	اسفناج خام	۲۰ برگ متوسط					
۸۴	اسفناج پخته	نصف لیوان					
۸۵	شلغم	۱ عدد متوسط					
۸۶	فلفل سیاه	۱ عدد متوسط					
۸۷	سس قرمز	۱ ق.غ					
۸۸	ترشی (با ذکر محتویات)	۱ ق.غ					محتویات:
۸۹	شور (با ذکر محتویات)	۱ ق.غ					محتویات:
۹۰	خیار شور	۱ عدد متوسط					
۹۱	طالبی	۱/۴ نوع متوسط					
۹۲	خربزه	۱ قاچ متوسط					
۹۳	هندوانه	۱ قاچ متوسط					
۹۴	گلابی	۱ عدد متوسط					
۹۵	زردآلو	۱ عدد متوسط					
۹۶	گیلاس	۱۰ عدد متوسط					
۹۷	سیب	۱ عدد متوسط					
۹۸	هلو	۱ عدد متوسط					
۹۹	شلیل	۱ عدد متوسط					
۱۰۰	گوجه سبز	۱ عدد متوسط					
۱۰۱	انجیر تازه	۱ عدد متوسط					
۱۰۲	انجیر خشک	۱ عدد متوسط					
۱۰۳	انگور	۱ خوشه کوچک					
۱۰۴	کیوی	۱ عدد متوسط					
۱۰۵	گریپ فروت	۱ عدد متوسط					
۱۰۶	پرتقال	۱ عدد متوسط					
۱۰۷	خرمالو	۱ عدد متوسط					
۱۰۸	نارنگی	۱ عدد متوسط					
۱۰۹	انار	۱ عدد متوسط					
۱۱۰	خرما	۱ عدد متوسط					
۱۱۱	آلو (زرد و قرمز)	۱ عدد متوسط					
۱۱۲	آلبالو	۱۰ عدد متوسط					

۱۱۳	توت فرنگی	۳ عدد متوسط					
۱۱۴	موز	۱ عدد متوسط					
۱۱۵	لیمو شیرین	۱ عدد متوسط					
۱۱۶	لیمو ترش	۱ عدد متوسط					
۱۱۷	آب گریپ فروت	۱ لیوان					
۱۱۸	آب پرتقال	۱ لیوان					
۱۱۹	آب سیب	۱ لیوان					
۱۲۰	آب طالبی	۱ لیوان					
۱۲۱	ذغال اخته	۱ لیوان					
۱۲۲	آناناس تازه	۱ لیوان					
۱۲۳	آناناس کنسرو	۱ لیوان					
۱۲۴	کشمش	۱ ق.غ					
۱۲۵	گرمک	۱کاسه ماست					
۱۲۶	توت تازه	۱۰ عدد متوسط					
۱۲۷	توت خشک	۲۰ عدد متوسط					
۱۲۸	برگه هلو	۱۰ عدد متوسط					
۱۲۹	برگه زردآلو	۱۰ عدد متوسط					
۱۳۰	زیتون سبز	۱۰ عدد متوسط					
۱۳۱	کمپوت میوه جات (با ذکر نوع و مقدار)						نوع: مقدار:
۱۳۲	روغن نباتی جامد	۱ ق.غ					
۱۳۳	روغن مایع	۱ ق.غ					آفتابگردان ذرت سویا
۱۳۴	روغن زیتون	۱ ق.غ					
۱۳۵	پیه	۱تکه متوسط					
۱۳۶	روغن حیوانی	۱ ق.غ					
۱۳۷	سس مایونز	۱ ق.غ					
۱۳۸	بادام زمینی	۲۰ عدد					
۱۳۹	بادام	۱۰ عدد					
۱۴۰	گردو	۱عدد مغز کامل					
۱۴۱	پسته	۱۰ عدد					
۱۴۲	فندق	۱۰ عدد					

۱۴۳	تخمه (کدو، آفتابگردان، هندوانه)	۱کاسه ماست خوری					
۱۴۴	قند یا شکر پنیر	۱۰ حبه					
۱۴۵	شکر	۱ ق.م					
۱۴۶	عسل	۱ ق.م					
۱۴۷	مربا (با نوع)	۱ ق.غ					
۱۴۸	نوشابه	۱ بطری					
۱۴۹	شیرینی خشک	۱ عدد متوسط					
۱۵۰	شیرینی تر	۱ عدد متوسط					
۱۵۱	گز	۱ عدد متوسط					آردی لقمه‌ای
۱۵۲	آبنبات	۱ عدد					
۱۵۳	سوهان	۱ قطعه					
۱۵۴	پفک	۱ بسته					
۱۵۵	شکلات	۱ عدد					
۱۵۶	کرم کارامل	۱ ق.غ					
۱۵۷	چای	۱ لیوان					
۱۵۸	نمک	۱ ق.غ					
۱۵۹	آبگوشت (فقط آب)	۱ لیوان					یددار غیریددار
۱۶۰	چیپس	۱ بسته					
۱۶۱	قهوه	۱ لیوان					
۱۶۲	آبلیمو	۱ ق.م					
۱۶۳	نبات	۱ تکه متوسط					
۱۶۴	قارچ پخته	نصف لیوان					
۱۶۵	حلوا خانگی	۱ ق.غ					نوع:......
۱۶۶	حلوا شکری	۱/۴ عدد					
۱۶۷	نقل	۱۰ عدد					
۱۶۸	پیراشکی	۱ عدد					
